# Calcitonin gene-related peptide-targeting drugs and Raynaud’s phenomenon: a real-world potential safety signal from the WHO pharmacovigilance database

**DOI:** 10.1186/s10194-022-01424-w

**Published:** 2022-05-03

**Authors:** Alexandre O. Gérard, Diane Merino, Elise K. Van Obberghen, Fanny Rocher, Alexandre Destere, Michel Lantéri-Minet, Milou-Daniel Drici

**Affiliations:** 1 Department of Pharmacology and Pharmacovigilance Center of Nice, University Hospital Center of Nice, Nice, France; 2 Department of Nephrology-Dialysis-Transplantation, University Hospital Center of Nice, Nice, France; 3 Laboratory of Molecular Physio Medicine (LP2M), UMR 7370, CNRS, University Côte d’Azur, Nice, France; 4 Pain Department and FHU InovPain, University Hospital Center of Nice and Côte d’Azur University, Nice, France; 5 Migraine and Trigeminal Pain, UMR 1107, INSERM, Auvergne University, Clermont-Ferrand, France

**Keywords:** CGRP-targeting drugs, CGRP receptor antagonist antibody, Gepants, Migraine, CGRP receptor, Calcitonin gene-related peptide, Raynaud’s phenomenon, Pharmacovigilance, Adverse drug reaction

## Abstract

**Background:**

Migraine is responsible for significant disability and societal burden. Recently, drugs targeting the calcitonin gene-related peptide (CGRP) pathway raised new hopes. CGRP, a potent vasodilator, plays a key role in the pathogenesis of migraine attacks. The deficiency of CGRP is involved in Raynaud’s phenomenon, which consists of abnormal vasoconstriction of the digits. We aimed to assess the potential association of Raynaud’s phenomenon with CGRP-targeting drugs, analyzing real-world data from the World Health Organization (VigiBase®).

**Methods:**

We queried all reports of Raynaud’s phenomenon involving a CGRP-targeting drug. We sought disproportionate reporting of Raynaud’s phenomenon with these drugs. For this purpose, we relied on the calculation of the Information Component (IC). A positive lower end of the 95% confidence interval (CI) of the IC defines a statistically significant association. As migraine patients are prone to Raynaud’s phenomenon, we also calculated the IC of Raynaud’s phenomenon with CGRP-targeting drugs compared to 5HT1_B/D_ agonists (triptans), and beta-blockers used in the treatment of migraine.

**Results:**

Overall, 99 reports of Raynaud’s phenomenon involving CGRP-targeting drugs have been yielded in VigiBase®. The most reported CGRP-targeting drug was erenumab, with 56 reports (56.6%). The median time to onset was 84 days. No fatality was notified, but one patient suffered from gangrene and extremity necrosis. As a whole, CGRP-targeting drugs were significantly associated with Raynaud’s phenomenon, with an IC of 3.3 (95%CI: 3.0–3.5). There was a disproportionate reporting of Raynaud’s phenomenon with CGRP-targeting drugs compared to triptans (IC 0.4; 95%CI: 0.1–0.6) and to beta-blockers (IC 0.5; 95%CI: 0.2–0.7) as well.

**Conclusions:**

There is a significant disproportionality signal of Raynaud’s phenomenon with CGRP-targeting. This signal stands out when CGRP-targeting drugs are compared to other drugs used in patients with migraine. This study is limited by missing data in pharmacovigilance reports. CGRP-targeting drugs may be subject to Weber effect and reporting bias. Nonetheless, CGRP blockade might be the last straw that disrupts the physiological balance of vascular response in patients at-risk of Raynaud’s phenomenon. Pending further data regarding vascular safety of CGRP-targeting drugs, caution is warranted in these patients.

## Background

Migraine is a primary headache with complex pathophysiology involving trigeminovascular system activation [1, 2]. Migraine afflicts almost 15% of the population [3] and is responsible for significant disability and substantial societal burden [4]. Treatments for migraine patients can be divided into two categories, abortive treatments that relieve headaches, and prophylactic therapy that reduces the frequency of migraine attacks. Unfortunately, drugs used in migraine prevention had so far a limited success, in terms of efficacy as well as patient adherence, due to their side effects [5].

Recently, a new paradigm-shifting class of drugs targeting the calcitonin gene-related peptide (CGRP) pathway has revolutionized migraine treatment [6, 7]. CGRP is the main neuropeptide released by the trigeminal nerve, whose signaling may be a key mechanism underlying the pathogenesis of migraine attacks, as CGRP is a potent vasodilator [8]. Therapeutic strategies to damper CGRP signaling include monoclonal antibodies directed against CGRP (fremanezumab, galcanezumab, eptinezumab), or the CGRP receptor (erenumab), and gepants which are small molecule antagonists of the CGRP receptor (rimegepant, ubrogepant, atogepant) [2, 9]. CGRP-targeting drugs are considered to be effective and generally safe but there are still uncertainties [2, 10, 11].

Due to their mechanism of action, CGRP-targeting drugs could theoretically induce adverse drug reactions (ADRs) underlain by impaired arterial vasodilation [12]. It is known that patients with migraine are at increased risk of vascular events [13–15]. Yet, there is still limited knowledge regarding the vascular outcomes of CGRP-targeting drugs [12, 16]. The fact that patients with cardiovascular risk factors are often excluded from trial participation led the European Medicines Agency (EMA) to mention such an “important potential risk” in the Risk Management Plan of CGRP-targeting drugs, pending further pharmacovigilance data [17–19].

The deficiency of CGRP is believed to play a role in Raynaud’s phenomenon [20, 21]. Raynaud’s phenomenon is underpinned by a local defect in vascular response. It consists of abnormal vasoconstriction in response to various conditions, like cold temperatures or emotional stress. The consequence is sharply demarcated color changes of the skin of the digits. Raynaud’s phenomenon often accompanies migraine [22, 23]. Cases of Raynaud’s phenomenon induced or aggravated by CGRP-targeting drugs have recently been reported [24–26].

We aimed to assess the potential association of Raynaud’s phenomenon with CGRP-targeting drugs, relying on real-world data issued from the pharmacovigilance database of the World Health Organization (WHO).

## Methods

### Data source

The WHO Safety Database (VigiBase®) is managed by the Uppsala Monitoring Center (UMC) [27]. Since 1967, VigiBase® gathers Individual Case Safety Reports issued from the national pharmacovigilance networks of more than 172 countries. These post-marketing reports originate from healthcare professionals, patients, as well as pharmaceutical companies. The anonymity of both patients and reporters is preserved. Each report contains administrative information (country, reporter qualification), characteristics of the patients (sex, age), drugs (indication, start and end dates, dose, concomitant drugs), and ADRs (effects, seriousness, onset, outcome).

### Query

All CGRP-targeting drugs belong to the N02CD class in the Anatomical Therapeutic Chemical (ATC) classification system: atogepant, eptinezumab, erenumab, fremanezumab, galcanezumab, olcegepant, rimegepant, telcagepant, ubrogepant, vazegepant. VigiBase® was queried for all reports containing the Preferred Term (PT) “Raynaud’s phenomenon” registered until January 31, 2022, and involving a CGRP-targeting drug (N02CD). In the Medical Dictionary for Regulatory Activities (MedDRA, version 24.1 [28]), the PT is defined as the distinct descriptor for a single medical concept [29]. To take into account coding heterogeneity, VigiBase® was also queried for all reports belonging to the High Level Term (HLT) “Peripheral Vasoconstriction, necrosis and vascular insufficiency” (which includes the PT “Raynaud’s phenomenon” inter alia) with a CGRP-targeting drug. Quantitative variables were described in terms of medians with interquartile ranges (IQR) and/or minimum-maximum ranges (min-max). Qualitative variables were described as numbers and proportions.

### Disproportionality analysis

Disproportionality is a case/non-case analysis used to detect pharmacovigilance signals [30, 31]. If the proportion of reports with a specific ADR and a given drug (cases) is greater than the proportion of reports with the same ADR and other drugs (non-cases), an association between this drug and the ADR is suggested. Disproportionality can be assessed by the Information Component (IC), derived from a Bayesian confidence propagation neural network [32]. The IC is a tool validated by UMC. It compares observed and expected numbers of reports with an ADR-drug combination. This tool allows earlier and more specific detection of potential pharmacovigilance signals compared to the other measures, such as the reporting odds ratio. A positive lower end of the 95% confidence interval (CI) of the IC is the common threshold used in statistical signal detection at UMC.

In this study, we used disproportionality to detect whether Raynaud’s phenomenon was reported differentially with CGRP-targeting drugs, as compared to all other combinations of ADRs and active ingredients in VigiBase®. Specifically, we calculated the IC for the combination of each CGRP-targeting drug with Raynaud’s phenomenon. As a sensitivity analysis, the same analysis was performed with the HLT “Peripheral Vasoconstriction, necrosis and vascular insufficiency”.

### Comparative disproportionality

As migraine patients are prone to Raynaud’s phenomenon, we sought whether CGRP-targeting drugs were still disproportionately involved in Raynaud’s phenomenon, when compared to 5HT1_B/D_ agonists and to beta-blockers (atenolol, metoprolol, nadolol, propranolol, timolol) used in the treatment of migraine. Indeed, 5HT1_B/D_ agonists (also known as triptans) and beta-blockers are widely used in patients suffering from migraine. Furthermore, both classes are known to induce Raynaud’s phenomenon per se [33–36]. This comparative disproportionality was calculated by the IC [37, 38]. This additional disproportionality analysis aimed to mitigate the impact of potential confounding factors in patients with migraine and to increase the specificity of any possible findings regarding CGRP-targeting drugs.

## Results

### Characteristics of the reports

As of January 31, 2022, 172 reports involving CGRP-targeting drugs in VigiBase® belonged to the HLT “Peripheral Vasoconstriction, necrosis and vascular insufficiency”, including 99 reports under the PT “Raynaud’s phenomenon” and 58 reports under the PT “Peripheral coldness”.

Regarding reports of Raynaud’s phenomenon, most patients were female (86, 92.5%), with a median age of 45 years (IQR: 35–57; min-max: 20–70). The United States issued most reports and physicians were the most frequent reporters (Table 1).

**Table 1:** Characteristics of the reports of patients with Raynaud’s phenomenon involving a CGRP-targeting drug

Characteristics	Number of reports (%)
Age	
18–44 years	29 (50.0)
45–64 years	27 (46.6)
65–74 years	2 (3.4)
Country	
United States of America	62 (62.6)
Italy	10 (10.1)
Germany	5 (5.1)
Spain	4 (4.0)
Ireland	4 (4.0)
Netherlands	4 (4.0)
Belgium	2 (2.0)
Switzerland	2 (2.0)
Norway	2 (2.0)
Austria	1 (1.0)
United Kingdom	1 (1.0)
Iceland	1 (1.0)
Sweden	1 (1.0)
Reporter qualification	
Physician	51 (51.5)
Pharmacist	3 (3.0)
Other Health Professional	16 (16.2)
Consumer	39 (39.4)

Reports were mainly related to CGRP monoclonal antibodies. The most reported CGRP-targeting drugs were erenumab with 56 reports (56.6%), galcanezumab with 28 reports (28.3), and fremanezumab with 13 reports (13.1%). Ubrogepant and rimegepant accounted for one report each (1.0%). There was no report with eptinezumab, atogepant, or CGRP blockade with a concomitant monoclonal antibody and small molecule CGRP receptor antagonist.

### Characteristics of reactions

The median time to onset was 84 days (IQR: 18–383). Median dose was 70 mg/month for erenumab (min-max: 70–140), 225 mg/month for fremanezumab (min-max: 225–225), and 120 mg/month for galcanezumab (min-max: 120–240). The ADR was deemed serious in 15 reports (15.2%), including 6 disabling and incapacitating cases. No fatality was notified, but one patient suffered from gangrene and extremity necrosis. The most frequently co-reported MedDRA terms were arthralgia (7 reports, 7.1%), alopecia (5 reports, 5.1%), condition aggravated (5 reports, 5.1%), constipation (4 reports, 4.0%), fatigue (4 reports, 4.0%), skin discoloration (4 reports, 4.0%), and weight increased (4 reports, 4.0%). Suspect co-reported active ingredients include propranolol in 3 reports (3.0%), rizatriptan and nadolol in 2 reports each (2.0%). CGRP antagonist was withdrawn in 23 reports (33.8%). Among 47 reports with available outcomes, Raynaud’s phenomenon did not recover in 31 reports (65.9%), was recovering in 3 reports (6.4%) and recovered in 13 reports (27.7%).

### Disproportionality analysis

As a whole, CGRP-targeting drugs were significantly associated with Raynaud’s phenomenon, with an IC of 3.3 (95%CI: 3.0–3.5). Specifically, Raynaud’s phenomenon was disproportionately reported with erenumab (IC IC 3.2; 95%CI: 2.8–3.5), galcanezumab (IC 3.2; 95%CI: 2.6–3.7) and fremanezumab (IC 3.2; 95%CI: 2.3–3.8). The IC025 of ubrogepant and rimegepant did not reach statistical significance.

The whole HLT “Peripheral Vasoconstriction, necrosis and vascular insufficiency” yielded an IC of 0.3 (95%CI: 0.1–0.5). The PT “peripheral coldness” did not reach statistical significance (IC 0.37; 95%CI: 0.0–0.72).

### Comparison with triptans and beta-blockers

As of January 31, 2022, 43 reports of Raynaud’s phenomenon were registered in VigiBase® with 5HT1 agonists. Given that 47,417 and 55,506 other ADRs were reported with 5HT1_B/D_ agonists and CGRP antagonists, respectively, the comparative IC of Raynaud’s phenomenon with CGRP antagonists was 0.4 (95%CI: 0.1–0.6).

Likewise, 142 reports of Raynaud’s phenomenon were registered with the beta-blockers atenolol, metoprolol, nadolol, propranolol and timolol. Given that 129,222 other ADRs were reported with those beta-blockers, the comparative IC of Raynaud’s phenomenon with CGRP antagonists was 0.5 (95%CI: 0.2–0.7).

## Discussion

Our analysis of the international pharmacovigilance database highlights a significant disproportionality signal of Raynaud’s phenomenon with CGRP-targeting drugs. This signal stands out even when CGRP-targeting drugs are compared to triptans. Yet, triptans are acute treatments of migraine, used in a similar population, and are known to induce Raynaud’s phenomenon [33, 34]. CGRP-targeting drugs are also more likely to be reported for Raynaud’s phenomenon than beta-blockers used as preventive treatments.

Women were represented in the overwhelming majority of reports, probably owing to the epidemiology of both migraine and Raynaud’s phenomenon [4, 39–42]. Furthermore, a hormonal influence on capsaicin-induced CGRP-mediated vasodilation of the skin has been described [43]. In our study, three CGRP monoclonal antibodies accounted for all but two reports. Erenumab, the front-runner of its class was the most frequently represented. Indeed, the oral small molecule CGRP antagonists were associated to Raynaud’s phenomenon in two reports only. This probably reflects the fact that ubrogepant and rimegepant are more recent, and less extensively used thus far. Moreover, ubrogepant and rimegepant are acute migraine treatments, so patients might be exposed to their potential ADRs for a shorter period, possibly decreasing the frequency of Raynaud’s phenomenon [44].

The involvement of the deficiency of CGRP in the pathogenesis of Raynaud’s phenomenon has been described as far back as the 1990s [20, 21]. These findings led to consider CGRP as a possible candidate to treat Raynaud’s phenomenon and systemic sclerosis [45–47]. In fact, CGRP receptor activation results in vasorelaxation and dilation of blood vessels [8, 48]. This mechanism likely underpins the involvement of CGRP-targeting drugs in Raynaud’s phenomenon. Accordingly, by decreasing CGRP release, triptans may alleviate migraine and in some cases induce Raynaud’s phenomenon [49, 50].

Yet, according to clinical trials, the cardiovascular safety profile of CGRP-targeting drugs is thus far reassuring [51]. The rate of vascular events between CGRP-targeting drugs and placebo-treated patients does not differ [12, 52, 53]. Nonetheless, real-life post-marketing pharmacovigilance data are extracted from a larger, non-selected, population of long-term treated patients. These data are paramount to detect a signal for potential ADRs, that might have escaped initial scrutiny [54].

In fact, in 2019, three cases of Raynaud’s phenomenon induced or exacerbated by CGRP monoclonal antibodies have been reported [24]. In addition, the safety of CGRP-targeting drugs among patients with Raynaud’s phenomenon has recently been assessed [25]. Microvascular complications occurred in 9 of 169 patients (5.3%), ranging from worsening Raynaud’s phenomenon to gangrene, requiring distal digit amputation. Broadly speaking, the long-term effects of CGRP blockade, especially in conditions of acute ischemia, still raise many questions [51, 55, 56].

The present study has several limitations. This signal, highlighted by the statistical analysis of quantitative data, needs further qualitative assessment. Despite the disproportionality analysis and the comparison with Raynaud’s phenomenon reported with triptans and beta-blockers, we cannot rule out the possibility that some reports were wrongly attributed to CGRP-targeting drugs. Available clinical data are too scarce to distinguish with certainty an aggravation of a preexisting Raynaud phenomenon from the new onset of a Raynaud phenomenon. Likewise, heterogeneity in the coding of outcomes prevents from precisely assessing the reversibility of Raynaud’s phenomenon. Indeed, some recoveries may apply to an isolated episode of Raynaud phenomenon, while others may relate to all Raynaud’s crises broadly. Another confounding factor may be that CGRP-targeting drugs are used in patients with severe migraines, possibly at higher risk of Raynaud’s phenomenon (even if no correlation has been described to date). Besides, our signal may be confounded by the Weber effect, whereby recent drugs are subject to a rise in ADR reporting during their first years of marketing [57, 58]. Healthcare professionals might also have been influenced by the notoriety bias due to increased awareness towards vascular safety of CGRP blockade [59]. Beta-blockers are not a perfect control group, as they are not exclusively used in patients with migraine. Head-to-head comparisons between drugs classes should not be extrapolated on. Pharmacovigilance studies cannot draw definite conclusions regarding the causal relationship between CGRP antagonists and Raynaud’s phenomenon. Nonetheless, the underlying mechanism appears plausible.

## Conclusions

CGRP blockade might be the last straw that disrupts the physiological balance of vascular response in patients at-risk of Raynaud’s phenomenon. Although uncommon, Raynaud’s phenomenon triggered or aggravated by CGRP blockade may lead to serious complications. Pending further long-term data regarding vascular safety of CGRP-targeting drugs, caution is warranted when considering the use of those promising drugs in patients at-risk of Raynaud’s phenomenon.

## Data Availability

The data that support the findings of this study are available from Uppsala Monitoring Center (UMC) but restrictions apply to the availability of these data, which were used under license for the current study, and so are not publicly available. Access to VigiBase® is available without fees to Dr. Fanny Rocher. Data are however available from the authors upon reasonable request and with permission of UMC.

## Abbreviations

ADR: Adverse Drug Reaction
ATC: Anatomical Therapeutic Chemical
CGRP: Calcitonin Gene-Related Peptide
CI: Confidence Interval
EMA: European Medicines Agency
HLT: High Level Term
IC: Information Component
IQR: InterQuartile Range
MedDRA: Medical Dictionary for Regulatory Activities
Min-max: Minimum-maximum
PT: Preferred Term
UMC: Uppsala Monitoring Center
WHO: World Health Organization
